# Maximizing Success: An Overview of Optimizing the Ovarian Tissue Transplantation Site

**DOI:** 10.5935/1518-0557.20240027

**Published:** 2024

**Authors:** Koray Görkem Saçıntı, Rowaida Sadat, Sinan Özkavukçu, Meltem Sonmezer, Murat Sönmezer

**Affiliations:** 1 Department of Obstetrics and Gynecology, Ankara University School of Medicine, Ankara, Turkey; 2 Division of Epidemiology, Department of Public Health, Hacettepe University Faculty of Medicine, Ankara, Turkey; 3 Ankara University School of Medicine, Ankara, Turkey; 4 University of Dundee, School of Medicine, Assisted Conception Unit, Postgraduate Medicine, Ninewells Medicine, Dundee, UK; 5 Ankara Trade Center, Private Office, Ankara, Turkey

**Keywords:** ovarian tissue cryopreservation, ovarian tissue transplantation, transplantation site, retroperitoneum

## Abstract

Ovarian tissue cryopreservation and transplantation (OTCT) has emerged in recent years as a potential method for reversing abnormal endocrine and reproductive functions, particularly in patients receiving gonadotoxic cancer treatments having longer survival rates. From its first rodent experiments to human trials, OTCT has evolved tremendously, opening new windows for further utilization. Since then, significant progress has been achieved in terms of techniques used for surgical removal of the tissue, optimal fragment size, freezing and thawing procedures, and appropriate surgical sites for the subsequent reimplementation of the graft. In addition, various approaches have been proposed to decrease the risk of ischemic injury, which is the leading cause of significant follicle loss during neo-angiogenesis. This review aims to discuss the pros and cons of ovarian and retroperitoneal transplantation sites, highlighting the justifications for the viability and efficacy of different transplantation sites as well as the potential advantages and drawbacks of retroperitoneal or preperitoneal area.

## INTRODUCTION

Numerous studies have been conducted recently to put new techniques into practice to preserve fertility and endocrine functions in patients undergoing gonadotoxic cancer treatment and menopause-related estrogen deficiency symptoms. These studies have been conducted in conjunction with increased life expectancy and survival rates in cancer patients (Hashim *et al*., 2016; Karacan *et al*., 2020; Kolibianaki *et al*., 2020). In recent years, ovarian tissue cryopreservation and transplantation (OTCT) has been among the most feasible and successful methods to preserve endocrine functions and fertility (Yding Andersen *et al*., 2019). Moreover, feasibility and effectiveness of utilizing OTCT as an elective procedure to delay menopause and subside menopause-related symptoms has taken increased attention (Oktay *et al*., 2021). With reported live births exceeding 150 with ovarian tissue transplantation as of 2021, it was declared no longer an experimental approach in 2012 by the American Society of Reproductive Medicine (Marin & Oktay, 2022; Practice Committees of the American Society for Reproductive Medicine & the Society for Assisted Reproductive Technology, 2013). In relation to safety, an increasing number of studies have consistently demonstrated a lack of noticeable disparity in chromosomal abnormalities, congenital malformations, and developmental concerns between human embryos examined and pregnancies arising from ovarian tissue cryopreservation (OTC), in comparison to natural conceptions or pregnancies achieved through alternative assisted reproductive technologies (ART) (Cobo *et al*., 2001; Noyes *et al*., 2009; Chung *et al*., 2013). However, with all increased success rates, transplantation techniques of the OTCT procedure are still yet to be defined regarding neovascularization injury, which remains the leading barrier to OTCT success.

## BASICS ASPECTS OF THE OTCT

OTC procedure includes surgical removal of ovarian tissue mainly by laparoscopic surgery, isolation of cortical tissue fragments by denuding of the ovarian stroma, and subsequent cryopreservation using slow freezing or vitrification. Several studies have shown minimal follicle loss during cryopreservation either by freezing or vitrification (Yding Andersen *et al*., 2019). The cryopreserved ovarian tissue has demonstrated to have a longevity up to 17-year and requires 3-6 months to re-establish full function following transplantation (Lotz *et al*., 2020; Ruan *et al*., 2020). The orthotopic sites of transplantation include pelvic side walls and the remaining ovary (Donnez & Dolmans, 2014; Andersen *et al*., 2008). On the contrary, heterotopic transplantation sites involve the rectus sheath muscle and brachioradialis fascia in the forearm. These alternative sites offer unique advantages, such as being minimally invasive not requiring general anesthesia, cost-effective, and easily accessible when required. However, they also present inherent limitations, encompassing suboptimal conditions for follicular development, uncertainties regarding graft longevity, a scarcity of pregnancy-related data, and variations in individual outcomes (Kim, 2012; Beckmann *et al*., 2017).

## COMPARISON OF ORTHOTOPIC AND HETEROTOPIC SITES OF OVARIAN GRAFT TRANSPLANTATION

**Figure 1:**
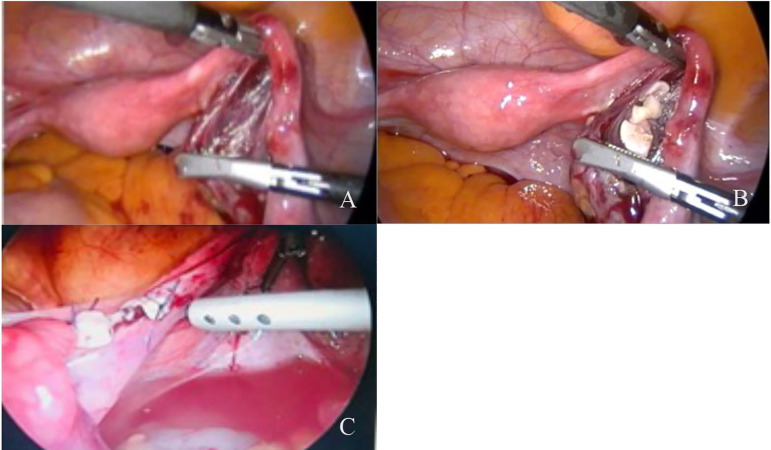
a. Creation of retroperitoneal pouch with laparoscopic instruments b. Transplantation of ovarian graft into the retroperitoneal pouch c. Transplantation of ovarian fragments onto the postmenopausal ovary.

Transplantation site is among the crucial factors for graft longevity and the success rate of the OTCT procedure. In decision-making, different aspects including the possibility of a natural conception, accessible sites for oocyte pick-up, the requirement of repeated transplantations, and the primary purpose of the procedure, namely whether it is for the fertility preservation, restoration of endocrine function or both, should be considered (Figure 1). The possibility of a natural conception is one of the critical strengths of orthotopic transplantation. In a study involving 95 orthotopic OTCT procedures with a mean age of 35±5.2 years at the time of transplantation, 21 pregnancies and 19 deliveries were reported. This yielded a pregnancy rate of 28% and a delivery rate of 33%, with variations depending on the age of the patients (Van der Ven *et al*., 2016).

In another study involving 20 cancer survivors who underwent OTCT after being thoroughly sterilized, 16 successful pregnancies and 10 deliveries were reported, demonstrating OTCT as an effective method to preserve fertility in patients undergoing gonadotoxic cancer treatment (Meirow *et al*., 2016). Although the risk of adhesion is reduced with laparoscopic surgery, transplantation in orthotopic sites is an invasive surgical technique that requires general anesthesia (Wallace *et al*., 2016; Wang *et al*., 2002). In addition, the limitation in the number of transplanted fragments due to the atrophic size of a postmenopausal ovary is another critical disadvantage. As opposed, orthotopic site is the natural location of ovarian cortical fragments, especially in terms of pressure and temperature issues.

On the other hand, heterotopic transplantation is considered a less invasive procedure, along with easy monitoring especially if there is a high risk of ovarian involvement, more space to harbor transplanted cortical tissue fragments, and giving the possibility of direct injection of novel agents to improve tissue survival (Kim, 2012). Since there is no need for general anesthesia graft removal is easier (Filatov *et al*., 2016). However, natural conception is not possible and due to pressure and temperature differences and absence of paracrine factors, follicle growth is usually compromised making the procedure not optimal when the primary concern is fertility (Amorim *et al*., 2013).

Various heterotopic sites have been evaluated both in animals and humans, such as rectus sheath, subcutaneous sites, brachioradialis fascia, breast tissue. Retroperitoneal/preperitoneal region is a feasible alternative to transplantation on the menopausal ovary (Kim *et al*., 2004; 2009). According to an experimental animal study, a considerable number of inactive primordial follicles were retained in the transplanted ovarian tissue, which indicated that the follicular survival rate following heterotopic transplantation of cryopreserved ovarian graft was high in mice (Luyckx *et al*., 2013). To improve tissue survival, albeit with limited success, various strategies have been practiced such as; supplementation of in freezing medium with vascular endothelial growth factor (VEGF) and growth hormone, transplantation to highly vascularized areas, supplementation with antiapoptotic agents, treating ovarian implants with platelet rich plasma and using decellularized ovarian scaffolds. A study by Oktay *et al*. (2016) highlighted the efficacy of ovarian tissue transplantation (OTT) using a human decellularized extracellular tissue matrix scaffold, robot-assisted minimally invasive surgery, and peri-operative pharmacological support, which resulted in a notable success rate, leading to the restoration of robust ovarian function in both patients in study following OTT.

## TISSUE ISCHEMIA AND NEO-ANGIOGENESIS: THE MAIN CHALLENGES FACING OTCT

Since the transplanted ovarian tissue requires four to five days to restore oxygen, this period of deoxygenation leads to severe follicle loss, thus constituting a significant challenge when considering OTCT (Van Eyck *et al*., 2009). Several studies have reported an average 65% rate of follicle loss owing to tissue ischemia. In a sheep model, a 65% loss of follicles after transplantation, with added extra loss of 7% as a result of thawing and cryopreservation, was reported (Baird *et al*., 1999). Likewise, neovascularization period was demonstrared as a leading causes of follicle loss during OTCT in another mouse model (Demeestere *et al*., 2009). Several factors are activated during the neovascularization period, such as inflammatory factors, oxidative stress, the transmission of macrophages, and other response elements, which cause cell destruction and cell death. Nevertheless, primordial follicles exhibit a notable tolerance to reduced oxygen perfusion for an extended period, attributable to their inherently low metabolic rate, which remains arrested at meiosis I (Kim *et al*., 2003; Vollmar *et al*., 1995).

As for neovascularization, it refers to the creation of new vascular network as a reaction to hypoxia and ischemic condition (Hassanpour *et al*., 2018; Nassiri & Rahbarghazi, 2014). This dynamic mechanism involves various factors, which can occur in pathological or physiological conditions (Rahbarghazi *et al*., 2014). It consists of several steps: endothelial cell migration, proliferation, and tubular morphogenesis (Redmer & Reynolds, 1996). Understanding the fundamental factors affecting the process, which especially involves VEGF, fibroblast growth factor (FGF), and platelet-derived growth factor (PDGF), is crucial in addressing approaches to increase graft longevity and decrease follicle loss (Hassanpour *et al*., 2017; Jabbour, 2009; Fraser & Duncan, 2009). The balance between proand anti-angiogenic factors essentially regulates the angiogenesis mechanism. VEGF and FGF are capable of promoting angiogenesis; however, the promising preclinical outcomes of VEGF treatment in animal experiments have not yet proved successful in humans (Ylä-Herttuala *et al*., 2007). Recent studies have indicated that incorporating VEGF and bFGF into the hydrogel of the transplanted tissue can enhance follicle survival (Takae & Suzuki, 2019; Li *et al*., 2016; Starke *et al*., 2011). Targeting these factors, which could create a potentiated environment, is essential to alleviate the increased follicular loss during the neovascularization period.

## RETROPERITONEUM OR PREPERITONEUM AS A PROPOSAL FOR BETTER NEO-ANGIOGENESIS OF GRAFT TISSUE

The retroperitoneum is a dense vascularized site that is hypothetically suitable for large volumes of ovarian tissue possibly with decreased risk of ischemia (Figure 1A, 1B and 1C). In an experimental study, ovarian tissues were transplanted autologously into the mesosalpinx, uterine serosa, omentum, and retroperitoneal iliac fossa (Suzuki *et al*., 2012). The contrast enhancement in the ovarian tissue implanted retroperitoneally into the left iliac fossa was visible using computed tomography scan. Growing follicles were also noted at the location designated as the iliac fossa transplant site, but no follicles were found in the ovarian tissue transplanted into the right mesosalpinx, despite the appearance of contrast enhancement.

In an experimental monkey study, the ovarian cortex was incised into cubes, and auto-transplantation was carried out into the omentum and retroperitoneal iliac fossa. The estrogen measure was 0 pg/mL after transplantation and elevated to 54.5pg/mL on day 15 and 130.1pg/mL on day 22. Accordingly, one mature follicle was detected by abdominal ultrasound in the retroperitoneal iliac fossa, and further was demonstrated in the left retroperitoneal iliac fossa at laparotomy (Igarashi *et al*., 2010).

According to Durando *et al*. (2012) study, the ovarian tissue was transplanted into the retroperitoneum, arbitrarily given N-acetylcysteine (NAC) subsequently to assess feasibility, follicles, and neo-angiogenesis. The number of antral and immature follicles and corpus luteum was higher in NAC-treated groups; furthermore, the number of blood vessels in the graft was also higher in the NAC-treated group along with decreased apoptosis (Durando *et al*., 2012). Besides being a dense neovascularization site, the adhesion risk after the incision is low. Between 2007 and 2016, 1302 patients diagnosed with cancer in the FertiProtekt network had their ovarian tissue biopsied for fertility preservation (Beckmann *et al*., 2018). The technique used in 61 transplantations (85.9%) was transplanting the ovarian tissue into a peritoneal sac. Adhesions were found to occur during the abdomen examination in 30 transplantations (42.3%) (Beckmann *et al*., 2018).

Oktay *et al*. (2019) first reported the feasibility and success of robotic assisted preperitoneal ovarian transplantation using a neovascularizing decellularized extracellular matrix scaffold. It is easier to perform oocyte pick-up abdominally from the preperitoneally transplanted frozen thawed ovary. Additionally, in their recent study, Oktay & Marin (2024) observed that orthotopic OTT yields enhanced gamete and embryo quality, along with comparable rates of endocrine function restoration and longevity. This finding suggests a favorable choice for individuals seeking conception. Furthermore, for those emphasizing the preservation of ovarian endocrine function, a less invasive option in the form of heterotopic OTT remains a viable alternative.

The abundancy of neoangiogenic factors in retroperitoneum accredits it as a promising site for ovarian transplantation. It has been recognized that endothelial Ca^+2^ signals are significant factors in vascular remodeling, that were regulated intracellularly by other pro-angiogenetic factors like VEGF (Feng *et al*., 2008). Transient Receptor Potential (TRP) channels are extensive cation channels localized in vascular endothelial cells that have long been linked to vascular remodeling and angiogenesis. TRP takes an essential role in intracellular signal transportation by moderating Ca^+2^ entry in response to factors like VEGF and FGF to promote angiogenesis or any minor changes in the configuration of the microenvironment (Feng *et al*., 2008). The investigations recommend that TRP channels, especially TRPV5 and TRPV6, are predominantly presented in the renal epithelium and are distinctly Ca^+2^ selective (Feng *et al*., 2008).

Early experiments demonstrated that endothelial TRPV1 might also be responsible for vascular remodeling (Negri *et al*., 2020). Subsequently, it has been illustrated that hypoxia elevated TRPV1 and TRPV4 activity in pulmonary artery vascular smooth muscle cells, thus escalating the Ca^+2^ reply to mechanical stimulation. These features strengthen the pro-angiogenic role of TRP and Ca^+2^ in the neoangiogenic process, which are abundant in retroperitoneal organs (Feng *et al*., 2008). Retroperitoneal transplantation has the additional remarkable benefit of preserving ovarian cortical tissue fragments if gonadotoxic therapy causes the ovaries to become atrophic. Moreover, a retroperitoneal or preperitoneal pocket may be a more suitable place due to its voluminous space for possible future use of adjunctive technologies such as co-transplantation of ovarian components with stem cells to improve vascularization and tissue engraftment, injection of growth factors, and/or angiogenic molecules.

## CONCLUSION

OTCT offers a new window into women’s reproductive health. Since its inception, assisted reproductive technologies have evolved significantly. As a result of various investigations on better transplanting sites and improvements in transplantation techniques, novel ideas have been proposed. When the pelvic architecture, micro-environment conditions, risk of adhesion, and neovascularization are considered, the retroperitoneal transplantation may constitute a robust alternative site for OTCT.
